# Ultrasound-Targeted Microbubble Destruction Improves the Migration and Homing of Mesenchymal Stem Cells after Myocardial Infarction by Upregulating SDF-1/CXCR4: A Pilot Study

**DOI:** 10.1155/2015/691310

**Published:** 2015-05-05

**Authors:** Lu Li, Shengzheng Wu, Zheng Liu, Zhongxiong Zhuo, Kaibin Tan, Hongmei Xia, Lisha Zhuo, Xiaojun Deng, Yunhua Gao, Yali Xu

**Affiliations:** ^1^Department of Ultrasound, Xinqiao Hospital, The Third Military Medical University, Chongqing 400037, China; ^2^Department of Blood Transfusion, Xinqiao Hospital, The Third Military Medical University, Chongqing 400037, China

## Abstract

Mesenchymal stem cell (MSC) therapy shows considerable promise for the treatment of myocardial infarction (MI). However, the inefficient migration and homing of MSCs after systemic infusion have limited their therapeutic applications. Ultrasound-targeted microbubble destruction (UTMD) has proven to be promising to improve the homing of MSCs to the ischemic myocardium, but the concrete mechanism remains unclear. We hypothesize that UTMD promotes MSC homing by upregulating SDF-1/CXCR4, and this study was aimed at exploring this potential mechanism. We analyzed SDF-1/CXCR4 expression after UTMD treatment in vitro and in vivo and counted the number of homing MSCs in MI areas. The in vitro results demonstrated that UTMD not only led to elevated secretion of SDF-1 but also resulted in an increased proportion of MSCs that expressed surface CXCR4. The in vivo findings show an increase in the number of homing MSCs and higher expression of SDF-1/CXCR4 in the UTMD combined with MSCs infusion group compared to other groups. In conclusion, UTMD can increase SDF-1 expression in the ischemic myocardium and upregulate the expression of surface CXCR4 on MSCs, which provides a molecular mechanism for the homing of MSCs assisted by UTMD via SDF-1/CXCR4 axis.

## 1. Introduction

Bone marrow-derived mesenchymal stem cells (MSCs) have the potential to regenerate cardiac myocytes and accelerate neovascularization after acute myocardial infarction (MI) [1, 2]. However, a significant barrier to the effective implementation of clinical MSC therapy is their inability to reach the target tissues with high efficiency [3, 4]. Multiple studies have shown that the biological effects generated by ultrasound- (US-) targeted microbubble (MB) destruction (UTMD) could help to improve the homing ability and transplantation efficiency of the MSCs following MI [5–7], but the UTMD mechanism remains unclear.

Research into the mechanism of UTMD-assisted MSC homing has been performed in recent years. Some studies showed that UTMD created pores in the capillary wall, which led to an increase in permeability of myocardial blood capillary [7, 8] and may help to promote the transmigration of MSC from the blood vessels to the target tissues. Some other studies found that local microenvironments after myocardial injuries played an important role in the chemotaxis and differentiation of MSCs [9]. UTMD could change the microenvironment within tissues caused by the local inflammatory response and offer the target tissues as cell niches that are more suitable for MSC homing [10]. In short, the present research on the mechanism of MSC migration and homing promoted by UTMD mainly focuses on the morphology and microenvironmental changes of the infarcted myocardial tissues in vivo. However, studies about the effects of UTMD on MSCs themselves are comparatively few. What are the impacts on MSC viability and migration after UTMD treatment? Furthermore, what expression changes in the gene or protein level occur within MSCs?

SDF-1 and its specific receptor, CXCR4, play a critical role in stem cell mobilization, chemotaxis, homing, and engraftment in the process of repairing infarcted hearts [11, 12]. SDF-1, secreted by cells within the ischemic myocardium or homing MSCs (paracrine effect), is the most important chemokine controlling MSC migration [13]. Transplanted MSCs can migrate toward inflammatory tissue along an SDF-1 concentration gradient by binding SDF-1 to CXCR4 on the surface of MSCs [14, 15]. Importantly, the vast majority of CXCR4 is located in the cytoplasm in a highly glycosylated form, with only a small proportion of culture-expanded MSCs expressing functional CXCR4 at the cell membrane [16, 17]. Thus, one effective way to promote MSC migration and homing is an upregulation of SDF-1 secretion in target tissues and the expression of CXCR4 on the surface of MSCs.

In this study, we hypothesize that SDF-1/CXCR4 may be the key factor in UTMD-assisted MSC migration and homing and that the biological effects of UTMD promote MSC homing possibly by increasing SDF-1 expression in the ischemic myocardium and upregulating the expression of CXCR4 on the surface of MSCs. To address this hypothesis, we prepared two kinds of conditional culture medium, one was supernatants collected from normal or hypoxia cultured medium of human cardiac myocytes (HCM) and human cardiac microvascular endothelial cells (HCMEC), the other was myocardial tissue extracts obtained from normal rats or MI model rats. We then detected the expression of SDF-1 and membrane CXCR4 after UTMD. In addition, we counted the number of homing MSCs labeled by green fluorescent protein and examined SDF-1/CXCR4 expression in the ischemic myocardium of MI rats that were treated with UTMD combined with intravenous infusion of MSCs. The goal of this study was to identify the potential mechanism whereby UTMD improves the migration and homing of systemically implanted MSCs following MI.

## 2. Materials and Methods

### 2.1. Experimental Animals

All Sprague-Dawley (SD) rats used in this study were provided by the Experimental Animal Center of the Third Military Medical University. This study was carried out in strict accordance with the recommendations in the Guide for the Care and Use of Laboratory Animals of the National Institutes of Health. All experiments were approved by the experimental animal ethics committee of Third Military Medical University. All surgery was performed under sodium pentobarbital anesthesia, and all efforts were made to minimize suffering.

### 2.2. Preparation of Human MSCs and Rat MSCs

Human MSCs were isolated from the bone marrow of three healthy donors, ages 26 to 28 years, with written informed consent according to the guidelines of the Third Military Medical University ethics committee, and the study followed the procedures approved by the local ethics committee. After culture and expansion, the human MSCs were defined as CD44, CD105 double-positive, and CD34, CD45 double-negative cells by using flow cytometry. Rat MSCs were isolated from the femur and tibia bone marrow of 3-4 week old Sprague-Dawley (SD) male rats. The phenotypic properties of rat MSCs were also identified with the positive surface markers CD29, CD44 as well as the negative surface markers CD34, CD45. Adipogenic and osteogenic differentiation were successfully induced in both human MSCs and rat MSCs. All cells were cultured with DMEM/F12 (Hyclone) medium containing 10% FBS (Gibco) at 37°C in 5% CO_2_. The third or fourth passage of MSCs was used for subsequent experiments.

### 2.3. Normoxic and Hypoxic Cell Culture of HCM and HCMEC

Fetal human cardiac myocytes (HCM) (Catalog number 6200) and human cardiac microvascular endothelial cells (HCMEC) (Catalog number 6000) were obtained from ScienCell Research Laboratories (San Diego, CA) and maintained in the culture medium supplied by the manufacturer. These cells were provided at passage 2 and were used between passages 3 and 6. When HCMs or HCMECs were approximately 80% confluent in culture, they were harvested and mixed in ratios of approximately 1 HCMEC to 4 HCMs. For normalization purposes, the mixed cells were plated in 12-well plates at a total number of 1 × 10^6^ cells/well (1.2 mL/well) in complete medium and cocultured for 12 h. Thereafter, the cells were subjected to either 24 h of normoxia (21% O_2_, 5% CO_2_) or of hypoxia (1% O_2_, 94% N_2_, 5% CO_2_). The supernatants were collected after treatment for subsequent studies in vitro.

### 2.4. Developing Rat Myocardial Infarction Model

A surgical myocardial infarction (MI) model was developed in SD male rats (6-7 weeks old, 200–250 g). Rats were anesthetized with 2% pentobarbital natrium (40 mg/kg) by abdominal injection. After endotracheal intubation, the animals were mechanically ventilated using a rodent ventilator (Taimeng Instruments, Chengdu, China). The heart was exposed, and the left anterior descending coronary artery (LAD) was ligated with a 6-0 suture at a level very close (1 mm) to the bottom of the left atrium. Evidence of successful coronary occlusion was confirmed by typical S-T elevation on an electrocardiogram and regional cyanosis of the distal myocardial surface.

### 2.5. Preparation of Normal Myocardial Tissue Extracts (NMTE) and Infarcted Myocardial Tissue Extracts (IMTE)

MI hearts were taken out two days later under full anesthesia. Areas of the ischemic myocardium were carefully dissected, minced, and transferred into a 5-mL tube containing PBS at a ratio of 500 mg : 1 mL. The mixtures were homogenized with a Pro200 homogenizer (Pro Scientific Inc., USA) and centrifuged at 4°C. The supernatant (meaning MTE) was collected and filtered using a 0.22 *μ*m filter (Millipore, USA). The normal MTE was obtained from normal SD rats without ligation of the LAD coronary artery. The preparation method was same as that of the infarcted MTE.

### 2.6. Ultrasound Device and Microbubbles

We used an Accusonic Plus (Metro Medical Australia Pty Ltd, Australia) therapeutic ultrasound system to perform UTMD in vitro. The US irradiation parameters were operated as follows: US frequency = 1 MHz; duty cycle = 10%; peak negative pressure = 0.6 W/cm^2^ (0.35 MPa); total irradiation time = 30 s; and dosage of MBs = 10^6^/mL. Lipid-coated MBs, named Zhifuxian, were prepared in our department as previously described [18] and were used for inducing acoustic cavitation. Zhifuxian was prepared by the lyophilization of a suspension of two lipids, 1,2-dipalmitoyl-sn-glycero-3-phosphoglycerol (DPPG) and 1,2-distearoyl-sn-glycero-3-phosphoethanolamine (DSPE), and agitated with perfluoropropane gas by a mechanical amalgamator. The mean particle diameter of the MBs was 2 *μ*m, and the concentration was 6–9 × 10^9^/mL.

### 2.7. In Vitro Experimental Grouping

Human MSCs were collected and seeded into 6-well plates at a density of 5 × 10^5^ cell/mL (2 mL/well) in fresh complete medium. After the cells adhered, 1 mL/well culture medium was removed and 1 mL conditional culture medium (described below) was randomly added to each well. The MBs diluted with saline were slowly added as well. The 6-well plates were rocked gently, and then, UTMD was performed with the transducer at the base of the plates. After UTMD, the MSCs were immediately placed back in an incubator. After 24 h of incubation at 37°C with 5% CO_2_, the MSCs in all groups were harvested for cell viability assessments and CXCR4 detection, and the supernatants from all groups were collected for SDF-1, VCAM-1, and ICAM-1 ELISA. Each experiment was independently repeated six times.

Two types of conditional medium were added into the culture medium of MSCs, and the in vitro grouping was divided into two corresponding parts.

(1) The conditioned medium was the supernatant collected from normal or hypoxia cultured medium of HCM and HCMEC.

The test groups were designed as follows: MSC cultured in ordinary DMEM/F12 medium group (control); MSC + normal supernatant group (M + NS); MSC + normal supernatant + UTMD group (M + NS + U); hypoxia supernatant group (HS); MSC + hypoxia supernatant group (M + HS); MSC + hypoxia supernatant + UTMD group (M + HS + U).

(2) The conditioned medium was the myocardial tissue extracts (MTE) obtained from normal rats or MI model rats.

The test groups were designed according to the following scheme: MSC cultured in ordinary DMEM/F12 medium group (control); MSC + normal MTE group (M + NMTE); MSC + normal MTE + UTMD group (M + NMTE + U); infarcted MTE group (IMTE); MSC + infarcted MTE group (M + IMTE); MSC + infarcted MTE + UTMD group (M + IMTE + U).

### 2.8. ELISA

The concentration of SDF-1, VCAM-1 or ICAM-1 in the supernatants was detected by ELISA. All ELISA kits were purchased from R&D Systems (Minnesota, USA) and performed according to the manufacturer's instructions. Particularly, the hypoxia supernatant group and the infarcted MTE group were the non-MSCs groups, and the samples needed to be diluted in the same manner as the other groups before ELISA testing for normalization purposes.

### 2.9. Assessment of Cell Viability

Viability of MSCs was measured using a CCK-8 kit according to the manufacturer's instructions (Beyotime, Shanghai, China). Briefly, all samples and blank control MSCs were harvested, seeded in 96-well plates with 2 × 10^3^ cells/well (100 *μ*L/well), and 10 *μ*L CCK-8 solution was subsequently added. After 2 h incubation, the absorbance of the cells in each well was measured at 450 nm using an enzyme-linked immunoassay analyzer (DU800, Beckman, USA). Cell viability (%) was calculated from the ratio between absorbance of the sample MSCs and the blank control MSCs.

### 2.10. CXCR4 Detecting

Flow cytometry was used to detect the expression of CXCR4 on the surface of MSCs using a PE-conjugated CXCR4 antibody (12G5, Santa Cruz, USA). PE-CXCR4 (20 *μ*L/sample) and the treated MSCs were incubated at 4°C for 30 min, washed, centrifuged, and resuspended in 500 *μ*L PBS for analyzing with a FACS caliber cytometer (Becton Dickinson, San Diego, USA).

Fluorescent real-time quantitative PCR analysis was performed to investigate the mRNA expression of CXCR4. After a 24 h incubation with the conditional medium, total RNA was extracted from the MSCs using Trizol Reagent (Invitrogen, USA) according to the manufacturer's instructions. The first-strand cDNA was then synthesized using a PrimeScript RT reagent Kit (Takara, Dalian, China). CXCR4 was amplified with the following primers: sense 5′-TTCTACCCCAATGACTTGTG-3′ and antisense 5′-ATGTAGTAAGGCAGCCAACA-3′. As an internal control, GAPDH was also amplified with the following primers: sense 5′-CATCTCTGCCCCCTCTGCTG-3′ and antisense 5′-GCCTGCTTCACCACCTTCTTG-3′. Real-time PCR was performed with an ABI Prism 7300 Sequence Detection System (Applied Biosystems, Foster City, CA) using a SYBR green master mix (Applied Biosystems). The ratio of CXCR4 mRNA expression relative to GAPDH mRNA expression was calculated. Furthermore, relative CXCR4 mRNA expression was normalized against that derived from the control group.

Western blot analysis was carried out to detect the expression of CXCR4 protein within MSCs. Treated MSCs were lysed and the total cellular proteins (40 *μ*g) were separated on a 12% SDS polyacrylamide gel and transferred to a PVDF membrane. The membrane was blocked with TBS + 0.1% Tween-20 (TBS-T) containing 5% skim milk for 2 h and then incubated overnight at room temperature with the rabbit anti-CXCR4 antibody (1 : 500, Abcam, Cambridge, UK) and finally incubated with goat anti-rabbit IgG antibody (1 : 100, Beyotime, Shanghai, China) for 1 h the next day. The protein bands were scanned and the protein expression of CXCR4 was quantified by densitometry and normalized against GAPDH.

### 2.11. Transwell Migration Assay

The test groups for the transwell migration assay were the same as described above, except for the deletion of the hypoxia supernatant group and infarcted MTE group. In addition, AMD3100, an inhibitor of SDF-1/CXCR4 axis, was used in the migration assay. Briefly, MSCs were harvested, resuspended in serum-poor medium, and seeded into the upper chamber (100 *μ*L, 1 × 10^6^/mL) of a 24-well transwell plate (Corning Costar, USA) with 8-*μ*m pore filters. In the groups requiring US treatment, human MSCs were seeded into 6-well plates first, subjected to UTMD, and then seeded into the upper chamber. In the group containing AMD3100, MSCs were pretreated by coculturing with AMD3100 (5 *μ*g/mL) (Sigma) for 30 min. The supernatants or myocardial tissue extracts assigned to the different groups were placed into each lower chamber (500 *μ*L). Following a 5 h incubation, the cells that did not migrate from the upper chamber were removed with a cotton swab. The cells on the lower surface of the membrane were stained with 0.1% (w/v) crystal violet solution for 20 min, and the number of them was calculated under a light microscope at 200x magnification in five randomly selected fields. Each experiment was repeated three times in duplicate.

### 2.12. MSC Labeling and Implantation

The rat MSCs and 64 MI model rats were used in the animal experiment. After 1 week of modeling, the MI rats were randomly divided into four groups: PBS (1 mL) infusion merely served as a control group (Con, *n* = 12), the MSCs infusion group consisted of 1 × 10^6^ cells suspended in 1 mL of PBS transplanted by caudal vein injection (MSC, *n* = 12), US irradiation and MB infusion (0.1 mL/kg) group (UM, *n* = 12), and UTMD and MSCs infusion group (MSC + UM, *n* = 12). A diagnostic US system (Siemens ACUSON S2000, USA) was used to perform UTMD. The US probe was placed on the anterior chest for 10 min at a harmonic frequency of 2.0/5.0 MHz with a mechanical index of 1.3. In the MSC + UM group, MBs were intravenously injected followed by the infusion of MSCs.

In addition, we labeled rat MSCs with green fluorescent protein (GFP) (Cyagen, Guangzhou, China) using GFP lentiviral transduction according to the manufacturer's protocol. The GFP-MSCs were used for tracking homing cells in MSC group (*n* = 8) and MSC + UM group (*n* = 8). After 48 h of cell transplantation, the survival of implanted cells was determined by the number of GFP-positive cells in frozen sections (8 *μ*m) made from MI hearts under a laser scanning confocal microscope. The number of GFP-positive cells was by randomly counting five fields from each rat of the two groups.

### 2.13. Western Blot and Immunohistochemistry (IHC)

The protein expression and distribution of SDF-1 and CXCR4 in the MI area were assessed by Western blot and IHC. Animals were sacrificed after seven days of treatment and the hearts were harvested quickly.

Subsets of the hearts were used for Western blot (described before), the rest of them were fixed in 4% paraformaldehyde, embedded in paraffin, and sectioned at 4-*μ*m thickness for IHC. The antibodies used in IHC were as follows: rabbit anti-SDF-1 primary antibody (1 : 50, Santa Cruz), rabbit anti-CXCR4 primary antibody (1 : 50, Abcam), and the goat anti-rabbit IgG (1 : 100, Beyotime) secondary antibody.

### 2.14. Statistical Analysis

All data are expressed as the mean ± SD. The independent *t*-test was applied for comparison of the number of GFP positive cells between two groups. One-way analysis of variance (ANOVA) was performed for statistical comparisons of all of the data, except for of the number of GFP positive cells. Statistical analysis was carried out using the SPSS 13.0 software, and *P* < 0.05 was considered statistically significant.

## 3. Results

### 3.1. Effect of UTMD on SDF-1 Secretion and MSC Viability

**Figure 1:**
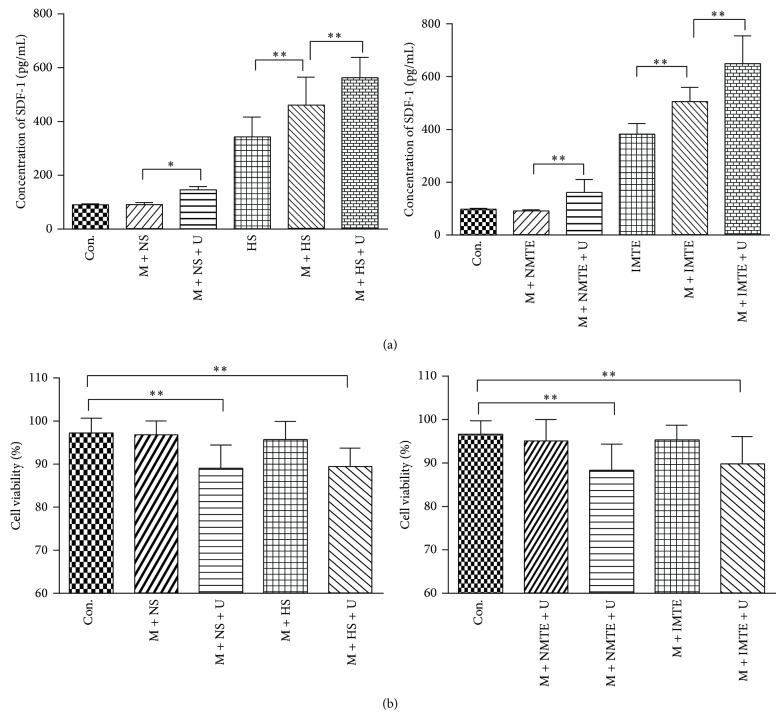
Effect of UTMD on SDF-1 secretion and MSC viability. (a) The assessment of the SDF-1 concentration for the various groups in the supernatant and MTE groups. (b) The assessment of MSC viability for the different supernatant and MTE groups. All values are expressed as the mean ± SD. ^∗^ *P* < 0.05; ^∗∗^ *P* < 0.01; *n* = 6.

In the supernatant groups, the concentration of SDF-1 in the M + NS + U group (145.87 ± 12.87 pg/mL) was increased by approximately 60% compared to the M + NS group (91.11 ± 8.96 pg/mL). The supernatants collected from hypoxia cultured medium of HCM and HCMEC (HS group) contained a certain amount of SDF-1 (344.89 ± 74.93 pg/mL). When the MSCs were cocultured with the hypoxia medium (M + HS group), the level of SDF-1 was increased by approximately 34% (462.51 ± 101.07 pg/mL). After UTMD treatment (M + HS + U group), the concentration of SDF-1 was further elevated by approximately 22% (563.75 ± 76.22 pg/mL). In the MTE fraction, the data showed a similar result (Figure 1(a)). In addition, in comparison to the control group, the viability of MSCs treated with UTMD decreased by approximately only 7%–9% in both the supernatant and the MTE groups (Figure 1(b)).

### 3.2. Effect of UTMD on the Expression of VCAM-1 and ICAM-1 In Vitro

**Figure 2:**
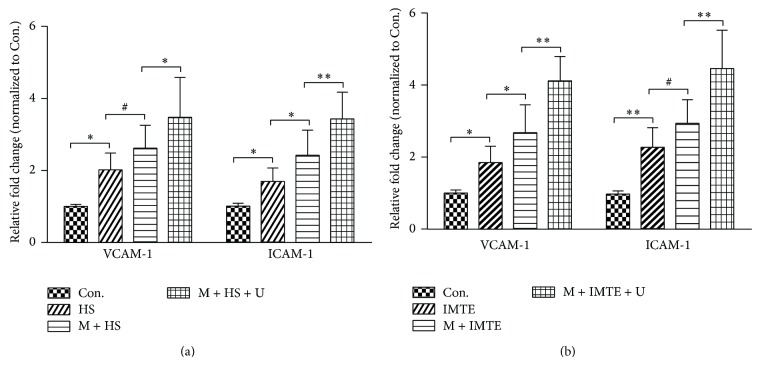
Effect of UTMD on the expression of VCAM-1 and ICAM-1. (a) The expression of VCAM-1 and ICAM-1 in the supernatant groups. (b) The expression of VCAM-1 and ICAM-1 in the MTE from each group. All values are expressed as the mean ± SD. ^∗^ *P* < 0.05; ^∗∗^ *P* < 0.01; ^#^ *P* > 0.05; *n* = 6.

To investigate the effects of UTMD on the upregulation of VCAM-1 and ICAM-1 expression, the supernatants obtained from the different groups were analyzed using an ELISA-based array, and the concentrations of the proteins were normalized against those derived from the control group. The data indicated that the hypoxia supernatants (HS group) or the infarcted MTE contained a large amount of VCAM-1 and ICAM-1 compared to those in the control group. UTMD increased the concentration of these proteins in the supernatant (Figure 2(a)) and MTE groups (Figure 2(b)) compared to the untreated groups.

### 3.3. CXCR4 Gene and Protein Expression In Vitro

**Figure 3:**
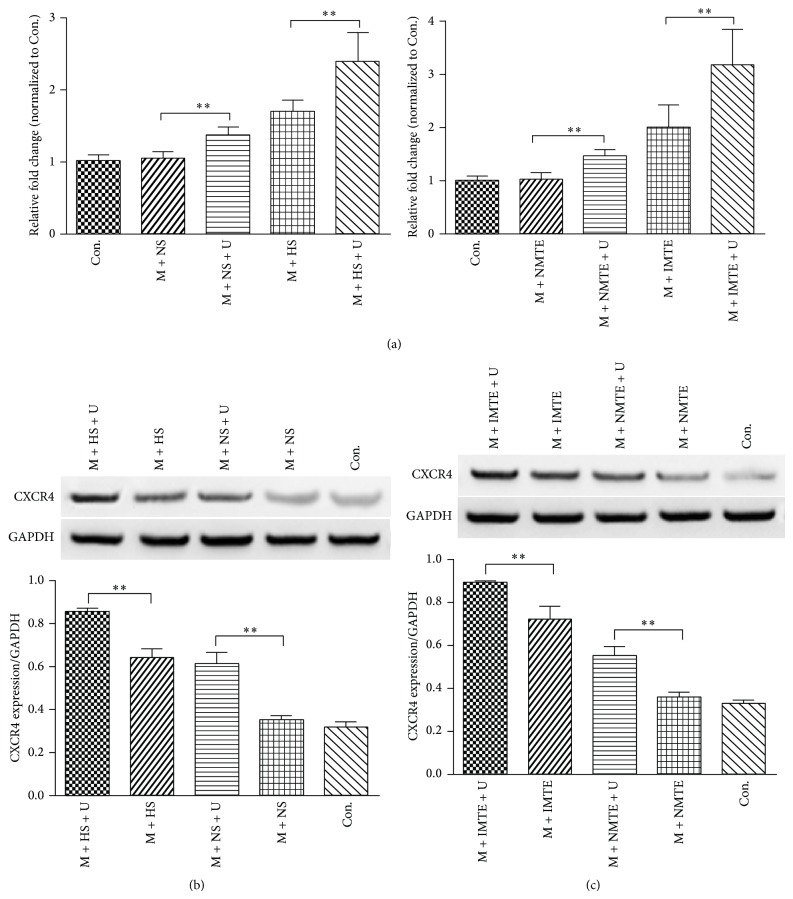
CXCR4 mRNA and protein expression in vitro. (a) Real-time PCR analysis of CXCR4 mRNA expression in MSCs from the different supernatant and MTE groups. The relative expression level was normalized against the control group, with GAPDH as an internal standard. ((b) and (c)) Western blot analysis for CXCR4 protein expression within MSCs from the different supernatant or MTE groups, respectively. The results from each group were normalized to GAPDH expression. All values are expressed as the mean ± SD. ^∗∗^ *P* < 0.01; *n* = 6.

The expression of CXCR4 mRNA was determined by real-time PCR at 24 h after coculture with conditioned medium (Figure 3(a)). In the supernatant groups, expression of CXCR4 mRNA was increased in the M + NS + U group (1.37 ± 0.11) compared to the M + NS group (1.05 ± 0.09) and reached the highest level in the M + HS + U group (2.39 ± 0.41). In the MTE groups, the results were similar to the supernatant.

Figures 3(b) and 3(c) showed the expression of the CXCR4 protein by Western blot. Compared to the untreated groups, the expression of CXCR4 protein was higher in the UTMD-treated group. The results were consistent with the CXCR4 mRNA levels and indicated that the UTMD-treated MSCs expressed more CXCR4 mRNA and protein than untreated MSCs.

### 3.4. Cell Surface Expression of CXCR4 with FCM

**Figure 4:**
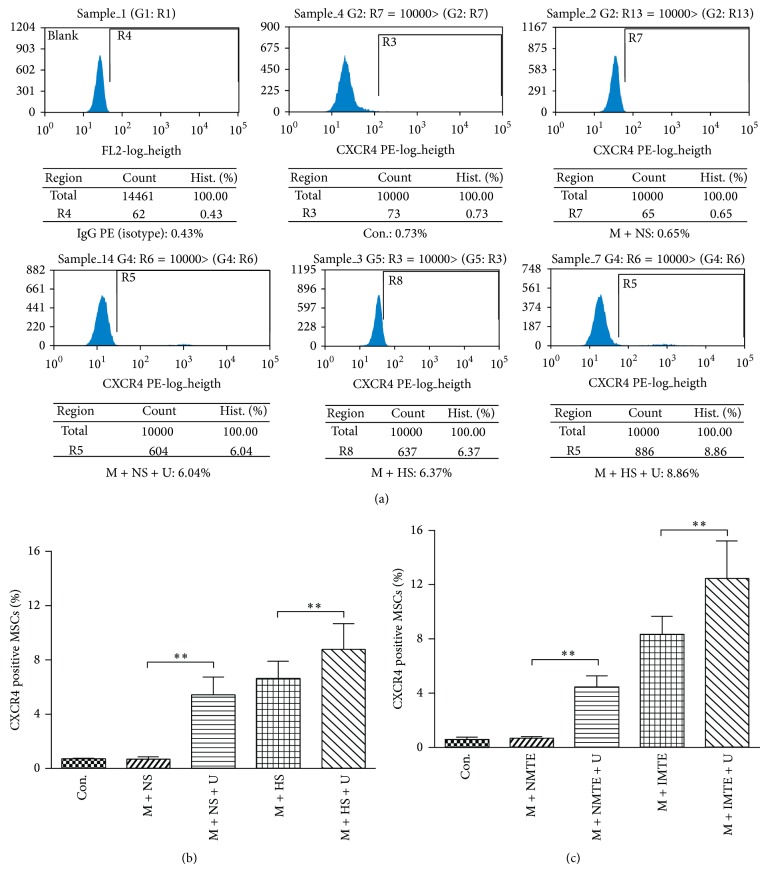
Cell surface expression of CXCR4 using FCM. (a) Representative examples of the membrane expression levels of CXCR4 on MSCs in the supernatant of the different groups. The percentage of MSCs positive for surface CXCR4 protein expression was valued as the number of cells expressing surface CXCR4/number of cells in total. (b) Quantification of cell surface CXCR4 protein expression in the supernatant groups. (c) Quantification of cell surface CXCR4 protein expression in the MTE groups. All values are expressed as the mean ± SD. ^∗∗^ *P* < 0.01; *n* = 6.

The cell membrane localization of CXCR4 was quantitatively evaluated by FCM (Figure 4(a)). The data showed that, for the supernatant groups, the percentage of cells expressing surface CXCR4 in the M + NS + U group (5.41 ± 1.29%) was 8.07-fold higher than the M + NS group (0.67 ± 0.17%) (Figure 4(b)), while the M + HS + U group (8.76 ± 1.94%) was 1.32-fold higher than the M + HS group (6.62 ± 1.27%) and 14.36-fold higher than the control group (0.61 ± 0.15%). The percentage of MSCs expressing surface CXCR4 in the M + NMTE + U group (4.48 ± 0.80%) was 6.79-fold higher than the M + NMTE group (0.66 ± 0.13%) (Figure 4(c)), while the M + IMTE + U group (12.45 ± 2.73%) was 1.49-fold higher than the M + IMTE group (8.34 ± 1.33%) and 22.23-fold higher than the control group (0.56 ± 0.19). The results indicated that, whether in the normal conditioned medium or in the hypoxia conditioned medium, UTMD could upregulate the expression of surface CXCR4 on MSCs in vitro.

### 3.5. In Vitro Migration Assay

**Figure 5:**
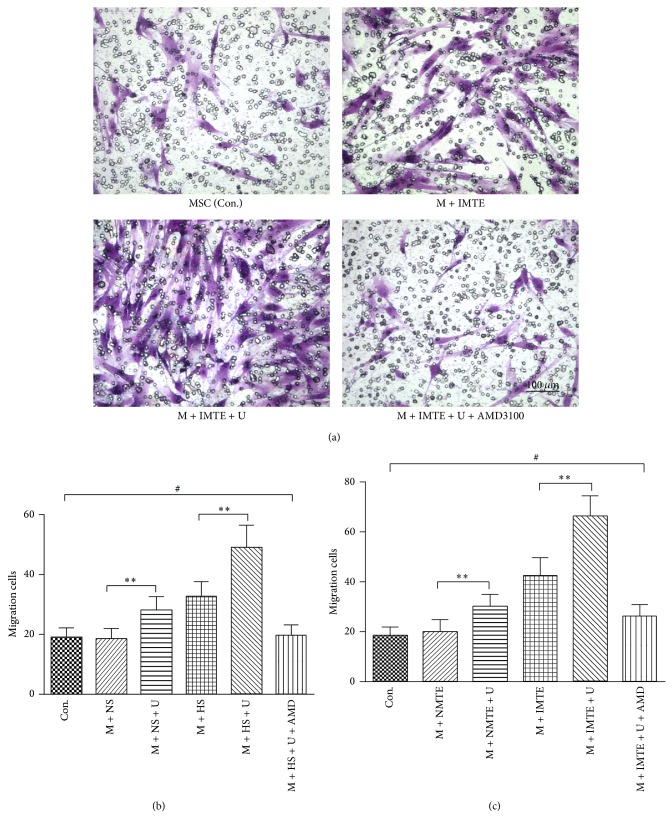
Effect of UTMD on the migration of MSCs. (a) Representative images of migrated MSCs in the MTE groups. Normal or infarcted myocardial tissue extracts were present in the lower chamber. The migrated cells were stained by crystal violet and observed under a light microscope. (b) Quantification of migrated MSCs in the supernatant groups. (c) Quantification of migrated MSCs in the MTE part groups. The scale bar represents 100 *μ*m. All values are expressed as the mean ± SD. ^∗∗^ *P* < 0.01; ^#^ *P* > 0.05; *n* = 3.

To detect the effect of UTMD on the migration of MSCs in vitro, a transwell migration assay was performed for the supernatant and MTE groups. As shown in Figure 5, the MSCs that migrated to the lower chamber were stained by crystal violet (Figure 5(a)), and the number of cells was recorded as follows: (supernatant groups, Figure 5(b)) (1) control group, 19.17 ± 3.06; (2) MSC + NS group, 18.67 ± 3.27; (3) MSC + NS + U group, 28.17 ± 4.45; (4) M + HS group, 32.83 ± 4.67; (5) M + HS + U group, 49.00 ± 7.46; (6) M + HS + U + AMD3100 group, 19.67 ± 3.39. The number of migrated cells in the MTE groups is shown in Figure 5(c): (1) control group, 18.50 ± 3.27; (2) MSC + NMTE group, 20.00 ± 4.77; (3) MSC + NMTE + U group, 30.33 ± 4.59; (4) M + IMTE group, 42.50 ± 7.01; (5) M + IMTE + U group, 66.17 ± 8.18; (6) M + IMTE + U + AMD3100 group, 19.50 ± 3.33. These data indicate that, in both the normal conditioned medium and in the hypoxia conditioned medium, the UTMD-treated MSCs migrated more efficiently compared to those in the untreated groups, which was blocked by AMD3100.

### 3.6. Identification of Homing MSCs

**Figure 6:**
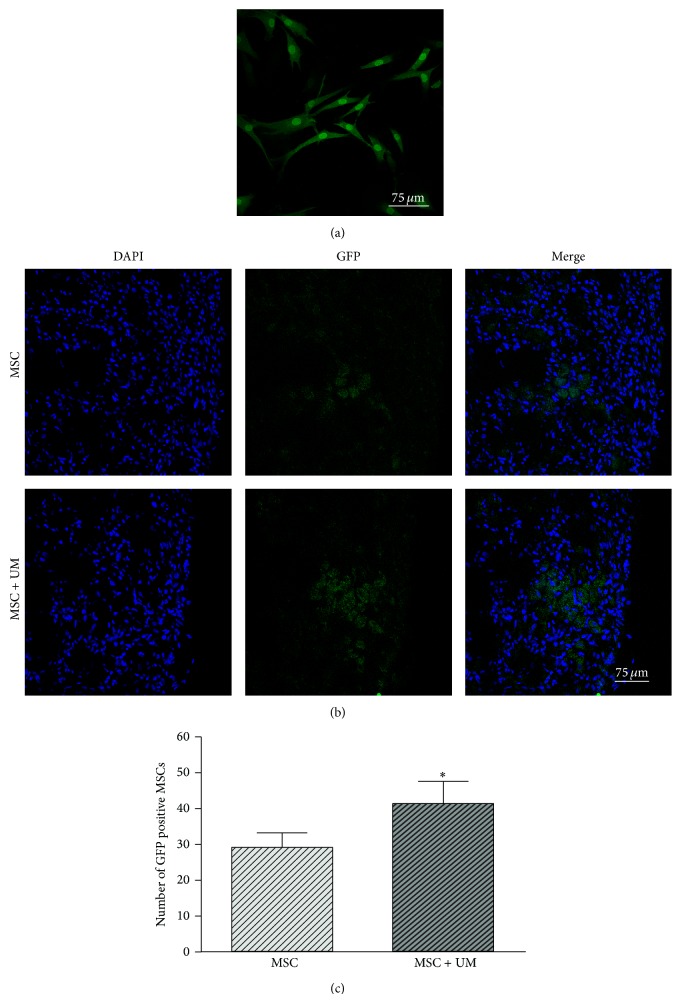
MSC labeling and the distribution of GFP-MSCs in the MI area. (a) MSCs were labeled with GFP; green fluorescence could be observed in both the cytoplasm and nucleus of almost all MSCs under a laser scanning confocal microscope. (b) GFP-positive cells of the MSC group and the MSC + UM group were distributed in MI areas 2 days after transplantation. (c) Quantification of the number of GFP-positive cells in the MSC group and the MSC + UM group. The scale bars represent 75 *μ*m. All values are expressed as the mean ± SD. ^∗^ *P* < 0.01 versus MSC group; *n* = 8.

Forty-eight h after GFP lentiviral transduction, bright green fluorescence could be observed in the cytoplasm and nucleus of almost all MSCs under a laser scanning confocal microscope (Figure 6(a)). We also confirmed the stable expression of GFP in subcultured MSCs. The GFP-positive MSCs were located in the ischemic myocardium and border zones but were essentially absent in the normal myocardium. The average number of GFP-positive cells in the MSC group and the MSC + UM group was calculated, and the results showed that there was a significant difference between the two groups. The number of GFP-positive cells in the MSC + UM group (41.27 ± 6.34) was significantly increased compared to the MSC group (29.23 ± 4.08) (Figures 6(b) and 6(c)).

### 3.7. Expression of SDF-1 and CXCR4 by IHC and Western Blot

**Figure 7:**
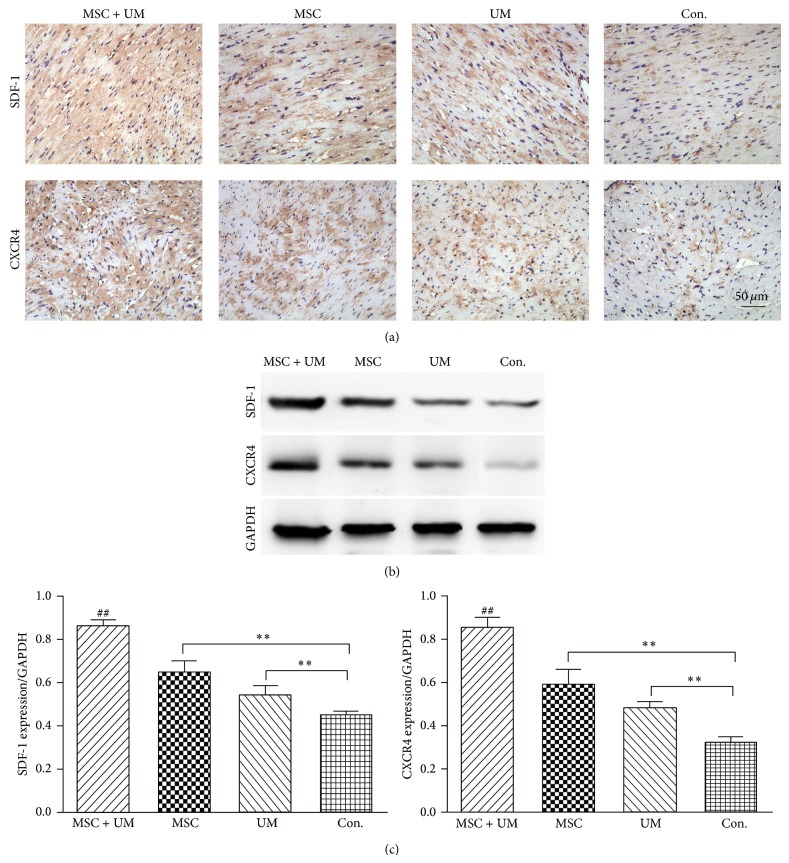
Expression of SDF-1 and CXCR4 by IHC and Western blot. (a) The immunohistochemistry results showed that the number of SDF-1- and CXCR4-positive cells was highest in the MSC + UM group. There were relatively fewer positive cells in the MSC group and UM group, and the control group had the lowest number of SDF-1- and CXCR4-positive cells. (b) Protein expression of SDF-1 and CXCR4 determined by Western blot in ischemic myocardium from four groups, with GAPDH as the internal control. (c) Quantification of SDF-1 and CXCR4 expression was performed by densitometric scanning. The scale bar represents 50 *μ*m. All values are expressed as the mean ± SD. ^∗∗^ *P* < 0.01; ^##^ *P* < 0.01 versus other groups; *n* = 6.

Immunohistochemical staining showed that SDF-1 and CXCR4 were predominantly localized to the cytoplasm in the ischemic myocardium and border area. The number of SDF-1- or CXCR4-positive cells was relatively smaller in the control group. More positive cells were observed in the MSC group and the UM group. The number of positive cells in the MSC + UM group was the largest, and in some slices, positive staining of SDF-1 and CXCR4 was distributed in a large quantity of cells, which took up almost the entire photograph (Figure 7(a)).

Western blot results showed that the level of SDF-1 in the ischemic myocardium was higher in the MSC group (0.65 ± 0.05) and the UM group (0.54 ± 0.05) compared to the control group (0.45 ± 0.02) and that the MSC + UM group (0.86 ± 0.03) had the highest levels compared to all other groups (Figures 7(b) and 7(c)).

## 4. Discussion

This study explored the mechanisms of UTMD-assisted migration and homing of MSCs to the ischemic myocardium through the SDF-1/CXCR4 axis. The in vitro results indicated that UTMD could promote SDF-1 secretion by MSCs, leading to an increased concentration of SDF-1 in the supernatants. Meanwhile, UTMD upregulated the percentage of MSCs expressing surface CXCR4 as well as the expression of CXCR4 gene and protein within MSCs. In vivo studies detected that, compared to the other groups, UTMD combined with intravenous infusion of MSCs resulted in greater numbers of homing MSCs and increased expression of SDF-1 and CXCR4 in the MI areas. It is generally known that MSCs have been widely applied in the treatment of various diseases because of their multipotent differentiation and immunomodulatory abilities [19, 20]. The treatment effect relies on the efficiency of MSCs homing to the target tissues. The process of MSC migration in vivo is complex, involving many types of cytokines, chemokines, adhesion molecules, and other proteins, but the SDF-1/CXCR4 axis is the most important molecular pathway for MSC migration and homing [21, 22]. Through in vitro and in vivo experiments, our results indicate that SDF-1/CXCR4 is also a crucial factor for promoting MSCs homing in UTMD, which can upregulate the expression of SDF-1/CXCR4 and improve the migration and homing abilities of implanted MSCs.

Our results in this work showed that higher concentrations of SDF-1 protein were detected in the supernatants of the UTMD-treated groups compared to the untreated groups (Figure 1(a)), whether in normal or hypoxia conditioned culture medium. This indicates that UTMD can boost SDF-1 secretion by MSCs. One of the reasons for this promoting effect may be the benefits from the biological effects of ultrasound. As a type of nonionizing energy, ultrasonic vibration can cause the movement of materials (such as cytoplasmic granules) within the tissues or cells, thus playing a role of subtle massage. Cavitation effects, generated by the destruction of MBs with US irradiation, has varying degrees of influence on the cell membrane fluidity, endocytosis, cytoskeleton, organelles, and other components, which are closely related to protein synthesis and the intercellular transport of particles and other materials [23]. The mechanism of the UTMD upregulation of SDF-1 expression in the ischemic myocardium may be more complex in vivo. The results in this study showed that higher levels of SDF-1 expression were present in the MSC group and the UM group compared to the PBS infusion group, and the MSC + UM group expressed the highest levels of SDF-1 compared to any other group (Figure 7). Upregulated expression of SDF-1 in the UM group may be induced by its biological effects. In the MSC group, increased expression may result from the paracrine activity of the implanted MSCs, while the combined effect of the UTMD-mediated response and the paracrine action of the homing MSCs may be responsible in the MSC + UM group.

Another important finding in this study is that compared to untreated MSCs, UTMD-treated MSCs had an increasing proportion of cells that expressed surface CXCR4 to different degrees, reaching a maximum value of 8.76 ± 1.94% in the M + HS + U group and 12.45 ± 2.73% in the M + IMTE + U group, which was 14.36-fold or 22.23-fold higher than the control group, respectively (Figure 4). As reported, only a very few culture-expanded MSCs expressed surface CXCR4 [16, 24]. Because recruitment of CXCR4 positive MSCs along the SDF-1 gradient plays a crucial role in cardiac recovery [25, 26], improving surface CXCR4 expression in the cultured MSCs are important for their therapeutic application. Until now, many methods have been reported to upregulate functional CXCR4 expression, including viral-mediated CXCR4 transduction [27], treating MSCs with a cytokine cocktail [28], cultivation of MSCs under hypoxic conditions [29], and surface modification by incorporating recombinant CXCR4 protein [30]. This study found that UTMD could increase the expression of membrane CXCR4 on MSCs as well. There are three potential mechanisms. First, the biological effect of UTMD may directly promote the transfer of abundant intracellular CXCR4 to the cell membrane. Second, the shockwaves and microjet flow generated by the destruction of MBs during cavitation results in the breakdown of the cell membrane and the reversible formation of tiny pores known as “sonoporation” [31], which effectively enhances the cell membrane permeability [32] and may provide a physical transfer channel for the transmembrane protein CXCR4. Third, UTMD can upregulate the expression of the CXCR4 gene and protein (Figure 3), and increased CXCR4 intracellular storage may also help the surface localization CXCR4.

The SDF-1/CXCR4 interaction, as a whole, is critical in MSCs migration and homing [33]. In our in vitro migration assay (Figure 5), UTMD-treated MSCs showed significantly increased migration ability compared to untreated MSCs, which may result from the upregulation of surface CXCR4 expression. In addition, the hypoxia conditioned medium combined with UTMD resulted in maximal MSC migration, which may benefit from the interaction of upregulated surface CXCR4 expression and increased SDF-1 contained in the hypoxia medium. AMD3100, an inhibitor of SDF-1/CXCR4, could block the promoting effects of UTMD and confirmed the key role of the SDF-1/CXCR4 interaction in UTMD-assisted MSC migration. As for studies in vivo, UTMD acts not only on the myocardium tissues but also on the MSCs gathered around the ischemic myocardium, leading to a promotion of the paracrine effect of MSCs and results in the increased secretion of SDF-1. The more SDF-1 is expressed in the MI areas, the more CXCR4 positive MSCs can migrate and home toward the SDF-1 gradient in the ischemic myocardium, and, further, the more SDF-1 can be secreted by the homing MSCs with UTMD to form a favorable cycle. In our in vivo results, we found that the number of GFP-positive MSCs homing to MI areas was significantly larger in the MSC + UM group than in the MSC group (Figure 6). Concomitantly, SDF-1/CXCR4 expression was higher in the former group (Figure 7). The results reaffirm that the biological effects of UTMD can facilitate the migration and homing of implanted MSCs; furthermore, it suggests that the SDF-1/CXCR4 interaction in vivo may play a continuous circular role in promoting MSC migration in a way different from the in vitro transwell migration assay.

VCAM-1 and ICAM-1, two types of adhesion molecules related to MSCs homing, were detected in vitro by ELISA, and the results indicate that UTMD can boost their secretion (Figure 2). In the in vitro study, these proteins were secreted by the MSCs at increased levels. If UTMD is applied in vivo, it will probably also promote other cells within the ischemic myocardium to secrete these adhesion molecules. Together with the SDF-1/CXCR4 interaction, increased VCAM-1 and ICAM-1 are advantageous to MSC migration and retention at the target tissues.

There are some other aspects needing to be explained in this study. First, hypoxia culture of HCM and HCMEC or infarcted myocardial tissue extracts served as the MI simulation in vitro. The former used the overwhelming majority of cells within the heart and may be helpful for normalization purposes because of the fixed number of these cells in each group. However, an advantage of the latter is that it is closer to the MI environment in vivo. In any event, the results acquired from the two groups of experiments are basically consistent. Second, as excessive ultrasonic irradiation often results in irreversible damages to cells and microvessels [34, 35], appropriate UTMD treatment is important for security considerations. In this study, we used the irradiation conditions referenced in the in vitro literature [34, 35], and the viability of MSCs only decreased by 7%–9% compared to the control group (Figure 1(b)). When UTMD was applied to rats, we used diagnostic US because lower US energy may be safer and more beneficial to the survival of implanted MSCs than therapeutic US. Certainly, some of the main parameters, such as acoustic intensity, treatment time, and MB dosage, must be optimized in future studies to achieve the purpose of more SDF-1/CXCR4 upregulation and less MSCs deaths. Third, though we have determined that UTMD can improve the expression of surface CXCR4 by flow cytometry, further studies using more-refined detection means (such as radioactive isotope localization) should be performed in future studies to directly confirm this result and further prove that the membrane CXCR4 comes from intracellular CXCR4 storage. Finally, current approaches to try to increase MSC therapeutic efficiency for cardiac repair, such as the use of UTMD, genetic manipulation, in vitro pretreatment of cells, or biomaterials, are confirmed by animal models in vast majority of studies. Despite positive results, rodent and even large animal models are just oversimplifications as compared to the human diseases. As far as UTMD, there is a certain distance from widespread clinical application because the real safety and effectiveness have not been assessed and perfected. Many subjects need to be improvement in future, including the timing of UTMD application, local and systemic effects from UTMD-assisted paracrine activities, and effect of UTMD on human heart function.

In summary, this study discusses how UTMD improves the migration and homing of MSCs to the ischemic myocardium. Our findings that UTMD can increase the expression of SDF-1 in MI areas and the surface levels of CXCR4 on MSCs shed light on one of the potential mechanisms underlying the upregulation of SDF-1/CXCR4. The present study provides a theoretical reference for UTMD-assisted MSC homing, and this new therapeutic approach may be promising in stem cell therapy on cardiovascular diseases.
